# Dantrolene as a Potential Strategy to Prevent Doxorubicin-Induced Cardiotoxicity: Mechanistic Promise and Clinical Prospects

**DOI:** 10.1016/j.jaccao.2024.12.002

**Published:** 2025-01-21

**Authors:** Itamar Braga Dias, Alexander H. Maass

**Affiliations:** University of Groningen, University Medical Center Groningen, Department of Experimental Cardiology, Groningen, the Netherlands

**Keywords:** anthracycline, Ca^2+^, calmodulin, cardiomyopathy, doxorubicin, heart failure, mechanisms, ryanodine receptor

**Figure:**
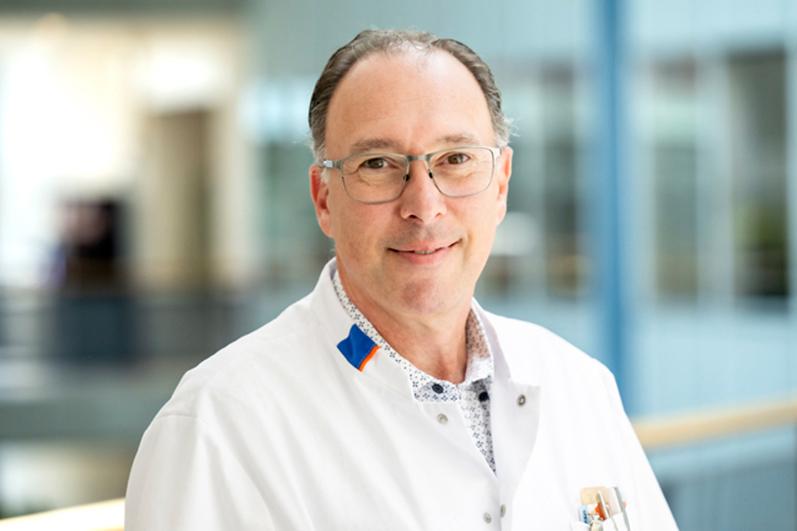

Doxorubicin’s efficacy as a chemotherapeutic agent is well-recognized; yet, its dose-dependent cardiotoxicity poses an ongoing challenge, particularly in patients undergoing long-term treatment. Addressing cardiotoxicity requires approaches that preserve doxorubicin’s anticancer effects while protecting cardiac function. In this issue of *JACC: CardioOncology*, Nakamura et al^1^ present an innovative study exploring dantrolene, a known ryanodine receptor (RyR2) stabilizer, as a potential cardioprotective agent against doxorubicin-induced cardiotoxicity (DIC). This study adds valuable insights into the mechanisms underlying DIC, particularly the role of RyR2-mediated calcium dysregulation, and suggests that dantrolene is a promising intervention in both in vitro and in vivo models. We outline key points regarding the study’s findings and their broader implications, noting considerations for future research.

The current study focuses on calcium dysregulation, identifying RyR2 destabilization as a major contributor to DIC. It is known that doxorubicin destabilizes RyR2, leading to pathological calcium leakage that triggers a cascade of reactive oxygen species (ROS) production, ER stress, and eventual cardiomyocyte death. By stabilizing RyR2, dantrolene effectively reduces this calcium leakage, which the authors associate with reduced oxidative stress and cellular damage in their murine model. This mechanistic focus on calcium regulation is particularly compelling, as it aligns with an emerging view of calcium homeostasis as a therapeutic target in cardio-oncology.^2^^,^^3^ Preclinical studies, such as those by Todorova et al,^4^ further support dantrolene’s cardioprotective potential, demonstrating its ability to mitigate doxorubicin-induced cardiotoxicity in animal models without compromising its anticancer efficacy. However, RyR2-mediated calcium leakage represents only one aspect of DIC’s multifactorial pathophysiology. Future studies that explore how RyR2 stabilization integrates with other potential interventions addressing mitochondrial dysfunction and inflammation may yield more comprehensive cardioprotective strategies.

The clinical variability of DIC presents an additional layer of complexity, particularly in cases where cardiotoxicity occurs at lower cumulative doses of doxorubicin. Prior research suggests that patient-specific factors, including genetic variability, may influence susceptibility to DIC. We have summarized some of the mechanisms but also protective and predisposing factors in Figure 1. Human induced pluripotent stem cell (iPSC)-derived cardiomyocytes have proven to be invaluable tools in modeling these individual differences.^5^ By replicating the responses of patient cardiomyocytes to anthracyclines, iPSC models enable a detailed understanding of how RyR2 dysregulation interacts with other molecular pathways, such as mitochondrial dysfunction and ferroptosis. Moreover, these models allow researchers to explore the genetic underpinnings of susceptibility, including sequence variations in calcium handling or oxidative stress response genes. This underscores the importance of integrating genomic data with iPSC-based platforms to refine cardioprotective strategies and advance precision medicine in cardio-oncology. Rather than a treatment targeting only one potential mechanism, strategies to address several pathways might be more promising. Exercise training is such an intervention that has been shown to reduce DIC in patients.^6^ In addition, exercise has many beneficial effects that could improve the outcomes of cancer patients even further.^7^

**Figure 1:**
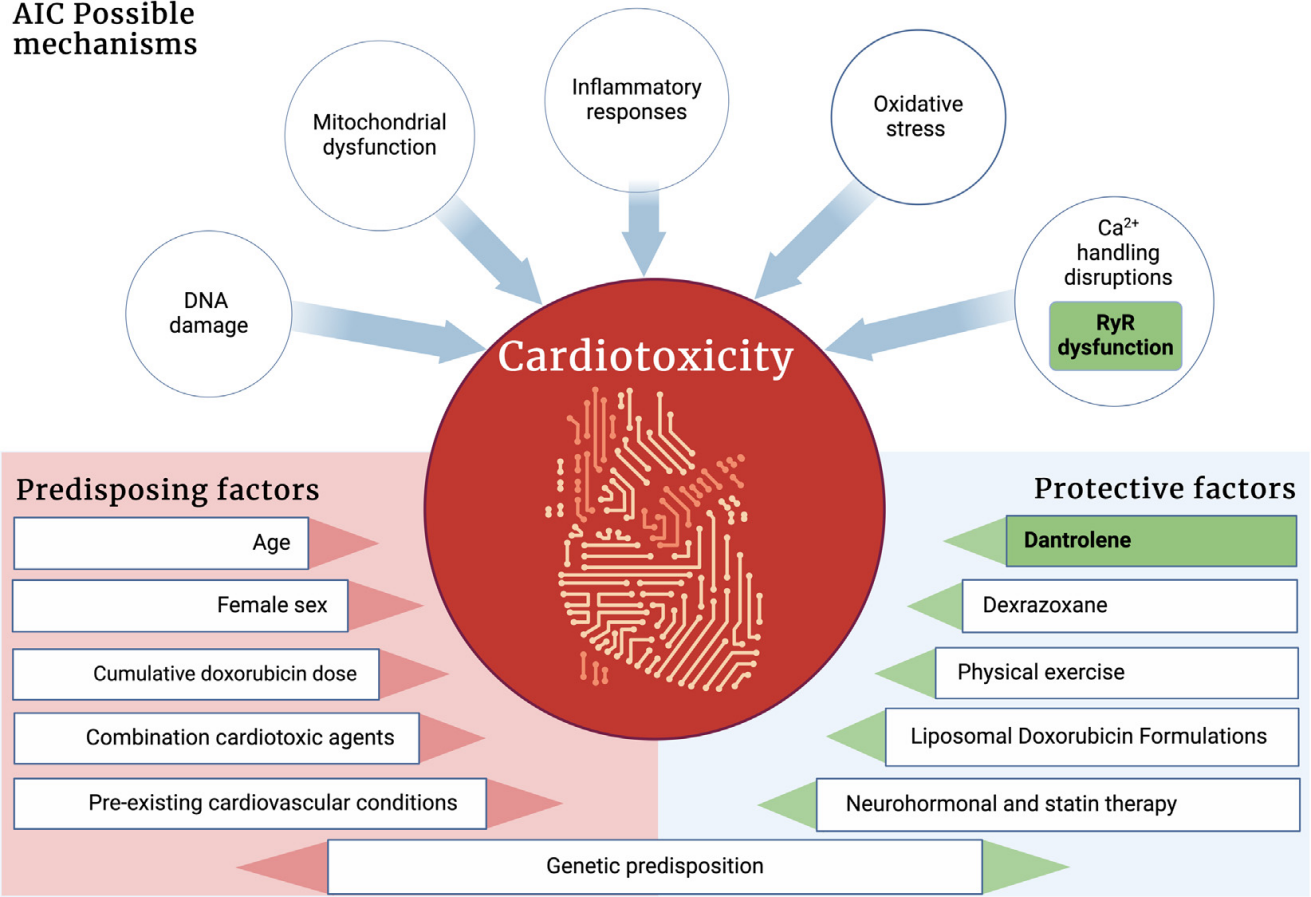
Mechanisms and Predisposing and Protective Factors for Anthracycline-Induced Cardiomyopathy The figure highlights some of the mechanisms that lead to anthracycline-induced cardiomyopathy (AIC). In addition, it shows protective and predisposing factors. RyR = ryanodine receptor.

Another strength of the current study is its repurposing of dantrolene, a drug with a long-established safety profile in treating malignant hyperthermia, which could allow faster translation to clinical trials in oncology. The authors report that short-term dantrolene use is sufficient for cardioprotection in their model, suggesting an attractive, limited-duration intervention that might easily fit into current doxorubicin regimens. However, this short-term application raises questions about long-term efficacy and safety. For patients requiring extended doxorubicin therapy, it remains unclear whether short-term RyR2 stabilization would be enough or if repeated or prolonged administration of dantrolene would be necessary. This highlights the need for clinical trials examining both acute and extended use in human populations to establish an effective and safe regimen for clinical use.

In addition, translating these findings from murine models to human applications introduces several complexities. Although the results provide a valuable proof of concept, variations in calcium channel regulation across species, coupled with genetic and sex-based differences, could influence how dantrolene functions in diverse patient groups. For example, sex-based differences in doxorubicin response have been documented, and exploring such variations in future human studies could help inform personalized approaches to dantrolene use.^8^ Rigorous human trials that include diverse demographics will be critical to understanding dantrolene’s clinical potential.

Finally, although the authors suggest that dantrolene mitigates the oxidative stress and endoplasmic reticulum stress linked to DIC, we must consider that RyR2 stabilization may address only part of the cardiotoxicity cascade. Doxorubicin-induced cardiomyopathy is multifaceted, with mitochondrial dysfunction and immune response activation also contributing significantly to cell injury. Although this study demonstrates RyR2 stabilization as an innovative approach, future research that examines additional or combined therapeutic targets may further strengthen our capacity to counter DIC’s complex pathophysiology.

In summary, Nakamura et al^1^ provide important mechanistic insights and introduce dantrolene as a novel, potentially repurposable intervention for DIC. Their study advances our understanding of calcium homeostasis in cardiotoxicity, emphasizing the role of RyR2 as a key therapeutic target. The integration of patient-specific platforms, such as iPSC-derived models, with clinical and preclinical findings, offers a promising pathway for understanding the variability in DIC and tailoring cardioprotective strategies to individual needs. Although the path to clinical application will require addressing several important questions, this work lays the groundwork for further exploration of targeted cardioprotective strategies in oncology. Moving forward, understanding where dantrolene fits within a comprehensive cardioprotection framework will be essential to ensure that cancer patients benefit from both effective cancer therapy and preserved cardiac health.

## Funding Support and Author Disclosures

The authors have reported that they have no relationships relevant to the contents of this paper to disclose.
